# Amphibian breeding phenology influences offspring size and response to a common wetland contaminant

**DOI:** 10.1186/s12983-021-00413-0

**Published:** 2021-06-25

**Authors:** Nicholas Buss, Lindsey Swierk, Jessica Hua

**Affiliations:** 1 Biological Sciences Department, Binghamton University, State University of New York, 4400 Vestal Parkway East, Binghamton, NY 13902 USA; 2 Environmental Studies Program, Binghamton University, State University of New York, 4400 Vestal Parkway East, Binghamton, NY 13902 USA

**Keywords:** Anthropogenic climate change, Amphibian, Contaminant tolerance, Phenology, Road salt

## Abstract

**Background:**

Increases in temperature variability associated with climate change have critical implications for the phenology of wildlife across the globe. For example, warmer winter temperatures can induce forward shifts in breeding phenology across taxa (“false springs”), which can put organisms at risk of freezing conditions during reproduction or vulnerable early life stages. As human activities continue to encroach on natural ecosystems, it is also important to consider how breeding phenology interacts with other anthropogenic stressors (e.g., pollutants). Using 14 populations of a widespread amphibian (wood frog; *Rana sylvatica*), we compared 1) growth; 2) tolerance to a common wetland contaminant (NaCl); and 3) the ability of tadpoles to acclimate to lethal NaCl exposure following sublethal exposure earlier in life. We evaluated these metrics across two breeding seasons (2018 and 2019) and across populations of tadpoles whose parents differed in breeding phenology (earlier- versus later-breeding cohorts). In both years, the earlier-breeding cohorts completed breeding activity prior to a winter storm and later-breeding cohorts completed breeding activities after a winter storm. The freezing conditions that later-breeding cohorts were exposed to in 2018 were more severe in both magnitude and duration than those in 2019.

**Results:**

In 2018, offspring of the later-breeding cohort were larger but less tolerant of NaCl compared to offspring of the earlier-breeding cohort. The offspring of the earlier-breeding cohort additionally were able to acclimate to a lethal concentration of NaCl following sublethal exposure earlier in life, while the later-breeding cohort became less tolerant of NaCl following acclimation. Interestingly, in 2019, the warmer of the two breeding seasons, we did not detect the negative effects of later breeding phenology on responses to NaCl.

**Conclusions:**

These results suggest that phenological shifts that expose breeding amphibians to freezing conditions can have cascading consequences on offspring mass and ability to tolerate future stressors but likely depends on the severity of the freeze event.

**Supplementary Information:**

The online version contains supplementary material available at 10.1186/s12983-021-00413-0.

## Introduction

Identifying how species are affected by anthropogenic climate change is an urgent challenge in ecology. One prominent ecological consequence of climate change is phenological shifts in critical life-history events associated with rising average temperatures and increased temperature variability [14, 53]. In temperate zones, warming temperatures have resulted in progressively earlier starts to spring breeding and migratory activities since the 1960’s [61]; this pattern is strong for species across taxa (e.g., marine animals – [52]; amphibians – [24]; plants – [3]; birds – [64]; insects – [65]; mammals – [48]). One cost of such temperature-induced shifts in the phenology of organisms are the risks associated with “false springs” [6, 37]. As climate change causes warmer winter temperatures, annual reproduction events may be prematurely cued (i.e., a false spring) exposing breeding individuals and/or their offspring at vulnerable early life stages to season-typical frost or freezing temperatures. The risk of false springs was identified early in our understanding of the effects of climate change on species’ phenology [13]. Since then, there have been numerous documented instances of freeze-induced plant death and damage due to premature budding and shooting (e.g., in the winters of 2007 [26] and 2010 [36]).

Of all the plant and animal taxa examined in a comprehensive meta-analysis, amphibians were identified as the taxon with the most advanced phenological shifts. Amphibian spring activities, on average, have shifted more than twice as early as that of trees, birds, or butterflies, and eight times as early as non-woody plants; the most extreme amphibian species exhibited early shifts of roughly 30 days/decade over the study period [50]. Notably, in a study on eastern North American amphibians, those that bred earliest (i.e., *Rana sylvatica, Pseudacris crucifer*) were also those shown to have the strongest climate-change related phenological shifts toward even earlier breeding [24]. In light of the increased variability in temperature and associated false spring events, early spring breeding amphibians are likely to fall victim to direct effects from post-emergence freezing. Though temperate adult amphibians have strategies to cope with freezing temperatures [60], repeated freeze-thaw cycles carry physiological costs [64] that can potentially lead to carry over effects that negatively affect offspring. Investigating potential carry over effects associated with phenological shifts have important implications for amphibian population persistence in the face of climate change.

Wildlife populations are also faced with threats from exposure to environmental contaminants [1]. Of growing concern is the salinization of freshwater systems, which has increased over the past several decades due to agricultural irrigation, coastal flooding and the application of road salts [12, 29]. Elevated salinities can result in direct mortality of freshwater species leading to deleterious outcomes for wildlife populations [29]. For wood frogs, salinization can negatively influence population demography [39], rates of growth and development via altered activity levels [27], and can reduce survival [9]. Unlike some contaminants, salts are conserved within the aquatic environment [21] and can accumulate over time. This issue is further compounded by the predicted effects of climate warming on wetland evaporation, where increased evaporation is predicted to reduce water volume, potentially further elevating salt concentrations in freshwater systems [20]. These concurrent issues are particularly problematic for spring-breeding amphibians, who breed following spring rains that can flush road salts applied during the winter months into breeding habitats [4], exposing amphibians to increased salinities across multiple life stages [2, 38]. Given the potential for increased freshwater salinization under climate change, there is a need to understand how altered temperature regimes and salts interactively effect wildlife health [45].

While salinization can negatively impact wildlife health, previous research suggests that wildlife populations are able to acclimate to contaminant exposure [31, 32]. For example, Wu et al. [67] found that pre-acclimation of larval Indian rice frogs (*Fejervarya limnocharis*) in saltwater concentrations of 7 g L^− 1^ for 48 h increased their survival to an otherwise lethal dose of saltwater at a concentration of 11 g L^− 1^. In addition to contaminant acclimation, research suggests that varying temperatures, such as those expected under climate change [45] also play a role in the ability of organisms to withstand contaminant exposure [43, 46]. For example, Kimberly and Salice [43] exposed *Physa polmin* snails to different temperature regimes (22 or 28 °C) and then measured their sensitivity to cadmium. No differences in cadmium toxicity were found when snails were acclimated to either temperature alone. However, when snails initially acclimated to one temperature were switched to the other (i.e., 22 to 28 °C, or 28 to 22 °C) at the time of cadmium exposure, snail mortality greatly increased [43]. Thus, contaminant acclimation and temperature variability can both individually alter the toxicity of contaminants to wildlife. However, as temperature variability under climate change scenarios can additionally influence the breeding phenology of organisms, there is a need to not only understand the influence of temperature on the acclimation of contaminant tolerance, but also how breeding phenology may interact with temperature to influence the ability of organisms to acclimate to contaminant exposure. Indeed, by potentially exposing adults to stressful freezing conditions prior to breeding, shifts in breeding phenology may lead to carry over effects that influence the ability for their offspring to tolerate or acclimate to contaminants.

Using a widespread early spring-breeding amphibian (wood frog; *Rana sylvatica*) as our model, the goal of this study was to evaluate how adult exposure to different temperature conditions due to phenological variation in breeding times influences offspring growth and performance (baseline tolerance to NaCl and ability to acclimate to NaCl). Specifically, across two breeding seasons, we compared the growth and performance of offspring from earlier-breeding parental cohorts vs. later-breeding parental cohorts. In both years, later-breeding parental cohorts experience an additional freeze-thaw cycle prior to breeding compared to earlier-breeding parental cohorts. Because repeated freeze-thaw cycles carry physiological costs [64], we predicted that tadpoles of parents that delay breeding in our study populations and encountered a winter storm would experience carryover effects making them smaller and less able to tolerate and less able to acclimate to NaCl exposure.

## Methods and materials

### Animal collection

**Fig. 1:**
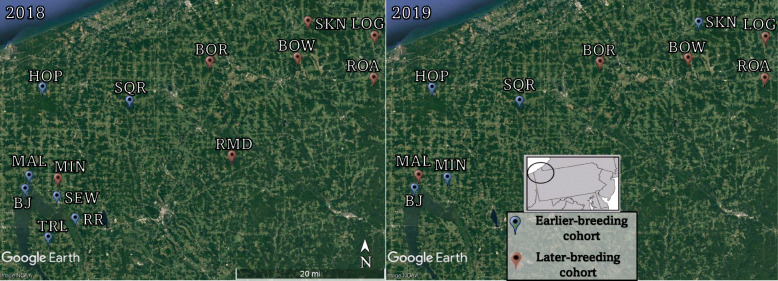
Map of wood frog source populations (Google Earth Pro version 7.3, Google, California, USA); Map of the wood frog source populations from western Pennsylvania, USA used in the experiments across the years 2018 and 2019. In-lay shows the broader geographic area of Pennsylvania, USA. The black circle within the in-lay shows the sampling region from which the populations were collected. Blue pins represent source populations of the earlier-breeding cohorts, and red pins represent source populations of the later-breeding cohorts

**Fig. 2:**
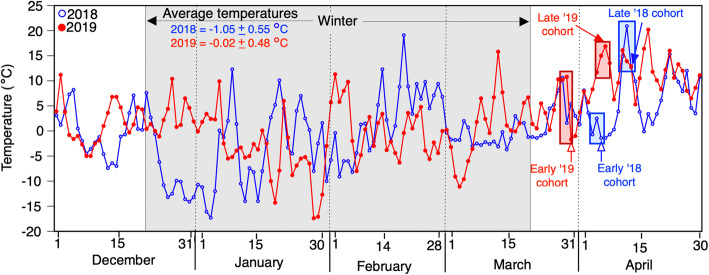
Temperatures and calling dates at wood frog collection sites; Average daily temperatures and wood frog egg collection days for early and late cohorts in 2018 (blue) and 2019 (red), respectively. Temperatures for the wood frog breeding sites were downloaded from Wunderground.com. Gray highlight represents days designated as winter for the northern hemisphere (December 21st-March 20th). Blue and red shaded boxes represent calling periods for 2018 and 2019 wood frogs (personal communications, J. Hua), respectively. Blue and red arrows represent collection dates for 2018 and 2019 wood frogs, respectively

To evaluate how differences in adult breeding phenology influences offspring growth and responses to NaCl, we compared wood frog tadpoles from populations that bred prior to freezing conditions (earlier breeding cohorts) vs. tadpoles from populations that bred after freezing conditions (later breeding cohorts) across two breeding seasons (2018 and 2019). In 2018, we collected egg masses from seven populations of wood frogs that bred on 6 April (earlier cohort) and egg masses from seven different populations that bred after a winter storm on 13 April (late cohort; Fig. 1; Fig. 2; Supplementary Materials Table 1). Similarly, in 2019, we collected egg masses from five populations of wood frogs that bred on 30 March (earlier cohort) and egg masses from each of 5 populations that bred after a winter storm on 7 April (late cohort; Fig. 1; Fig. 2; Supplementary Materials Table 1). All egg masses were collected from populations in western Pennsylvania, USA (Fig. 1; Supplementary Materials Table 1). The breeding window of wood frog populations in this region is characterized by breeding beginning in early March, continuing into April [25]. Thus, the seven-day difference in breeding times between the “earlier” and “late” populations represents a relatively significant difference in time of breeding given the short breeding window of wood frog populations in this region. Further, in addition to the seven-day difference in breeding dates, earlier- and- late-breeders are distinct from one another as only the later-breeding cohorts bred immediately after a winter storm. Thus, in both years, later-breeding parental cohorts experienced an additional freeze-thaw cycle prior to breeding compared to earlier-breeding parental cohorts.

To ensure broad sampling of each population, in both 2018 and 2019, we collected 10 partial egg masses from each site. To minimize any effect of environment on the egg masses, we collected egg masses within 48 h of oviposition.

### Natal pond conductivity

To account for variation that may arise in tadpole susceptibility to NaCl due to differences in natal pond exposure to NaCl, we measured pond conductivity at the time of egg collection. Water samples were taken from the edge and middle of each wetland, and specific conductance was measured (mean ± SE) using a YSI Multiparameter Sonde (Xylem, OH, USA). Specific conductance is a measure of water’s ability to pass electrical flow and is directly related to total ion concentration within the water [42]. Thus, our conductivity measurements do not distinguish between different salt ions within our water samples (e.g., chloride, sodium, magnesium, calcium, etc.). However, specific conductance has been shown to be strongly correlated with concentrations of sodium and chloride ions within bodies of water [39], particularly when road salts, such as NaCl, are thought to be a contributing factor to conductance [58]. Road salt applications within the counties that our populations reside in are applied largely with pure NaCl or salt brine (a mixture of NaCl and water [22];). Thus, our measurements of specific conductance are a reliable predictor of salinization resulting from inputs of NaCl across our pond sites.

### Experimental design

**Fig. 3:**
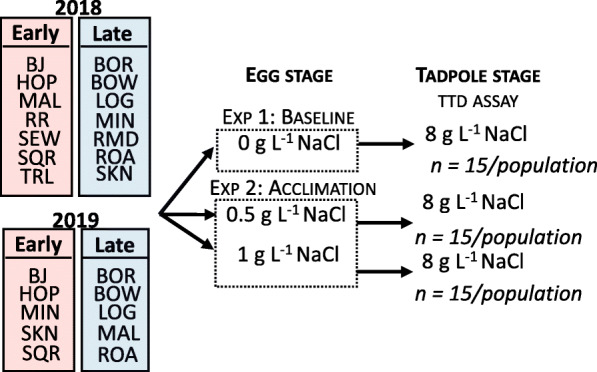
Baseline and acclimated tolerance experimental design schematic; Schematic of the experimental design for evaluating the baseline tolerance and acclimated tolerance of wood frogs to NaCl exposure

To assess mass, baseline tolerance to NaCl, and ability to acclimate to a lethal concentration of NaCl, we used a two-phase experimental design following Hua et al. [32]. We standardized our methodology across years and between earlier and late cohorts. In Phase 1, we employed a fully factorial design placing 50 embryos (Gosner stage 8; Gosner 1960) from each of the respective populations into 0 (background Cl^−^ concentration of 0.034 g L^− 1^), 0.50, or 1 g L^− 1^ NaCl solutions in 2 L plastic tubs, filled with 1.5 L of the respective Phase 1 pre-treatment solutions (Fig. 3). Once tadpoles reached the larval stage (Gosner stage 25), we measured the mass of a subset of tadpoles (see Supplementary Materials Table 2 for sample sizes) from all populations across the three treatment concentrations. We then initiated Phase 2, the time-to-death assay (hereafter TTD). Across both years and cohorts, all animals used in the mass measurements and TTD assay reached Gosner stage 25 within 12 h of one another. To start the TTD assay, we removed individuals from their respective Phase 1 exposure treatment and moved them to either a control (no salts added; *N* = 10) or a lethal NaCl treatment of 8 g L^− 1^ NaCl (*N* = 15). Experimental units were 100 mL cups filled with 80 mL of either control or NaCl treatment water, with a single tadpole/ experimental unit and were randomized across a single shelving unit. All experiments were carried out under identical temperature-controlled conditions (20 °C) between cohorts and years.

TTD assays are designed using concentrations of contaminants that cause mortality, while still allowing for the detection of variation in timing of mortality across treatments and populations [47]. Thus, we chose 8 g L^− 1^ NaCl, as it has been shown to be lethal to wood frog tadpoles [15, 56]. Additionally, 8 g L^− 1^ (~ 4.85 g L^− 1^ Cl^−^), albeit on the higher end of salt concentrations seen in natural systems, is environmentally relevant, with urban ponds, creeks, and rivers reaching concentrations of up to 13.50 g Cl^−^ L^− 1^ [20]. For information on the creation of the Phase 1 and Phase 2 NaCl solutions, please see Supplementary Materials. We measured the TTD of all individuals by assessing tadpole mortality every 2 hours until 100% mortality. In accordance with standard toxicology procedure, we did not feed any individuals during the TTD assay [49]. We euthanized all tadpoles that survived (i.e., control animals) using an overdose of unbuffered 5% MS-222 and then preserved all tadpoles used in the experiment in a 10% formalin solution.

### Statistical analysis

#### Natal pond conductivity

We used a linear mixed effect model to test for fixed effects of year, breeding cohort, and their interaction on pond conductivity. Following significant main effects, we conducted adjusted Bonferroni tests to evaluate pairwise comparisons.

#### Tadpole mass at Gosner 25

**Fig. 4:**
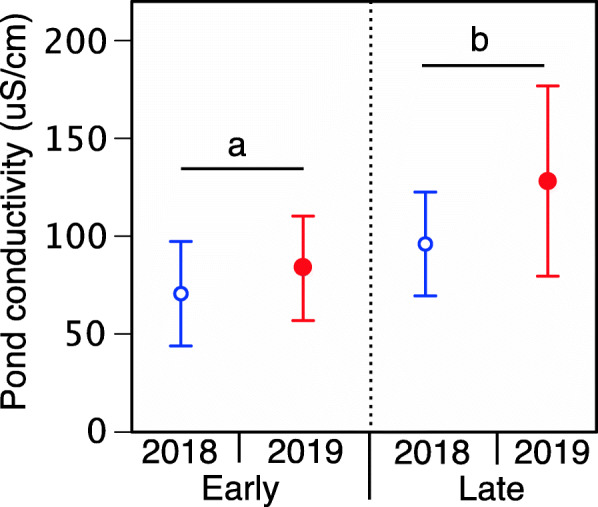
Average pond conductivity (± standard error) at wood frog sites; Natal pond conductivity for earlier vs. late breeding cohorts in 2018 and 2019. Different letters represent significance at *p* < 0.05

We used a linear mixed effect model to evaluate the mass of tadpoles from earlier vs. late cohorts after being reared in the various NaCl-acclimation treatments (0 g L^− 1^, 0.5 g L^− 1^, 1 g L^− 1^) in 2018 and 2019. We measured all tadpoles at Gosner stage 25 to control for differences in mass associated with development. Our model included cohort, acclimation concentration, year, and their interactions as fixed effects and pond conductivity as a random effect. We included pond conductivity in our model because the wood frog natal ponds varied in their conductivity (Fig. 4). For all significant main effects and interactions, we conducted Bonferroni-pairwise comparisons. Due to lost samples, we do not have mass data for tadpoles from the earlier-breeding cohort in 2018. These analyses were performed using SPSS version 26.

#### Baseline tolerance

We used Cox Mixed Effect models to compare the survival rates of tadpoles from earlier vs. late cohorts in 2018 and 2019. For baseline tolerance, we only considered tadpoles that were reared in the 0 g L^− 1^ NaCl acclimation treatment. In the first analysis, we included cohort, year, and their interaction as fixed effects and population and pond conductivity as random effects. Because there was a significant cohort*year interaction, we conducted two additional analyses, one for 2018 and one for 2019. These analyses were performed in R version 3.6. 2.

#### Effect of acclimation on mortality rate

We used Cox regression analysis to calculate beta coefficient (β), a measure of change in mortality risk for tadpoles exposed to the acclimation treatments relative to a one-unit change in mortality rate of tadpoles exposed to the control. For both 2018 and 2019, we calculated a separate β for each population by comparing the survival curve of tadpoles exposed to each acclimation treatment (0.5 g L^− 1^ and 1 g L^− 1^) to survival curves of tadpoles from the same population that were not exposed to NaCl (0 g L^− 1^). A negative β -value indicates that tadpoles exposed to the acclimation treatment had lower risk of mortality compared to tadpoles not exposed to the acclimation treatment. A positive β-value indicates that tadpoles exposed to the acclimation treatment had higher risk of mortality compared to tadpoles not exposed to the acclimation treatment. After calculating β as our metric for acclimation, we conducted a linear mixed effect model to compare acclimation in tadpoles (β) from earlier vs. late cohorts exposed to the various NaCl-acclimation treatments (0.5 g L^− 1^ vs. 1 g L^− 1^) in 2018 and 2019. Our model included cohort, NaCl-acclimation concentration, year, and their interactions as fixed effects and pond conductivity as a random effect. For all significant main effects and interactions, we conducted Bonferroni-pairwise comparisons. These analyses were performed using SPSS version 26.

## Results

### Natal pond conductivity

Using a linear mixed effect model, we found no effect of year (F_1, 7.8_ = 1.7; *p* = 0.23) or breeding cohort*year interaction (F_1, 8.5_ = 0.001; *p* = 0.98) on natal pond conductivity. However, there was a significant main effect of breeding cohort (F_1, 12.5_ = 12.5; *p* = 0.013) on natal pond conductivity. The conductivity of ponds for populations of tadpoles from later breeding cohorts was higher than the conductivity of ponds for populations of tadpoles from earlier breeding cohorts (Fig. 4).

To better understand whether patterns of natal pond conductivity influenced tadpole tolerance to NaCl, we conducted correlation analyses. We found no relationship between natal pond conductivity and tadpole tolerance to NaCl for tadpoles reared in 0 g L^− 1^ (*r* = − 0.22; *p* = 0.31), 0.5 g L^− 1^ (*r* = 0.34; *p* = 0.12), or 1 g L^− 1^ (*r* = − 0.18; *p* = 0.41). These data suggest that while pond conductivity differs between earlier and later cohorts, this variation does not appear to influence tadpole tolerance to NaCl. However, as a conservative approach, we include natal pond conductivity as a random factor in all analyses.

### Tadpole mass at Gosner 25

**Fig. 5:**
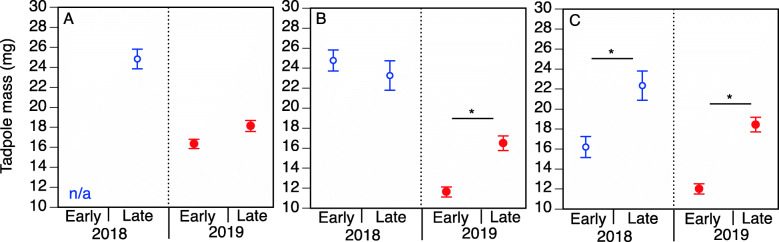
Influence of breeding phenology and NaCl exposure on wood frog mass; Average mass (± standard error) of Gosner stage 25 tadpoles for earlier vs. late breeding cohorts in 2018 (blue) and 2019 (red) when pre-exposed to (**a**) 0 g L^− 1^ NaCl, (**b**) 0.5 g L^− 1^ NaCl, or (**c**) 1 g L^− 1^ NaCl. Asterisks (*) denote significant differences (*p* < 0.05) between groups

Using a linear mixed effect model, we found a significant main effect of cohort (F_1,21.3_ = 5.0; *p* = 0.04), NaCl-acclimation treatment (F_2,599.9_ = 14.9; *p* < 0.001), and year (F_1,11.5_ = 27.6; *p* < 0.001) on tadpole mass at Gosner stage 25. Additionally, while there was no significant interaction between Cohort*year (F_1,21.8_ = 1.3; *p* = 0.25), there were significant interactions between Cohort*NaCl-acclimation (F_2,600.1_ = 10.2; *p* < 0.001), Year* NaCl-acclimation (F_2,600.5_ = 12.6; *p* < 0.001), and Year*NaCl-acclimation*Cohort (F_1,600.5_ = 6.6; *p* = 0.01). To facilitate the description of the significant interactions and in accordance with our a priori hypotheses, we separately describe the effect of earlier vs. late cohorts on tadpole mass at each year for each of the three NaCl-acclimation treatments. For tadpoles exposed to the 0 g L^− 1^ rearing condition, we do not have data for 2018 but for 2019, there was no significant difference in mass between earlier vs. late cohorts (*p* = 0.44; Fig. 5). For tadpoles exposed to the 0.5 g L^− 1^ rearing condition, in 2018, there was no significant difference in mass between earlier vs. late cohorts (*p* = 0.44; Fig. 5). However, in 2019, there was a significant difference in mass between earlier vs. late cohorts (*p* = 0.04; Fig. 5). Tadpoles from earlier breeding cohorts were smaller than tadpoles in later breeding cohorts. Finally, for tadpoles exposed to the 1 g L^− 1^ rearing condition, there was a significant difference in mass between earlier vs. late cohorts in both 2018 and 2019 (*p* = 0.004 and *p* = 0.009, respectively; Fig. 5).

### Baseline tolerance

**Fig. 6:**
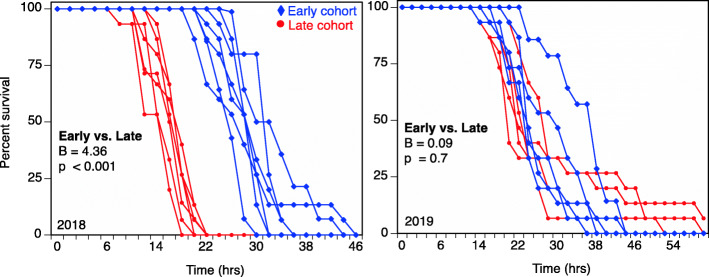
Influence of breeding phenology on wood frog NaCl tolerance; Baseline tolerance (average time to death ± standard error) to NaCl 8 g L^− 1^ NaCl) of wood frog tadpoles from parents in earlier (blue) vs. late breeding (red) cohorts in 2018 and 2019

In the overall Cox Mixed Effect model, while there was no effect of year (β = 0.02; *p* = 0.9), we found a significant effect of cohort (β = 5919.3; *p* < 0.001) and cohort*year (β = − 2.9; *p* < 0.001). To understand the interaction, we additionally conducted two separate Cox Mixed Effects analyses for 2018 vs. 2019. In 2018, we found a significant effect of cohort (β = 4.4; *p* < 0.001). Tadpoles from earlier breeding cohorts were significantly more tolerant to lethal concentrations of NaCl than tadpoles from later breeding cohorts. However, in 2019, we did not find a significant effect of cohort (β = 0.94; *p* = 0.7; Fig. 6).

### Effect of acclimation on mortality rate

**Fig. 7:**
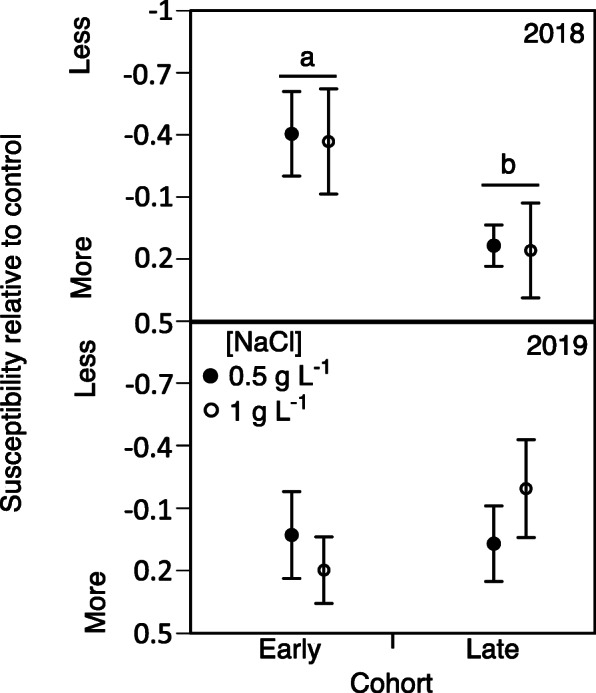
Influence of breeding phenology and NaCl exposure on wood frog acclimated tolerance; Acclimated tolerance (average β-coefficient ± standard error) to NaCl (8 g L^− 1^ NaCl) of wood frog tadpoles from parents in earlier vs. late breeding cohorts in 2018 (top) and 2019 (bottom) following an acclimation period in 0.5 g L^− 1^ or 1 g L^− 1^ NaCl. Different letters above groups denote statistical significance (*p* < 0.05)

Using a linear mixed effect model, we found no significant main effect of cohort (F_1,24_ = 1.2; *p* = 0.28), NaCl-acclimation treatment (F_1,24_ = 1.2; *p* = 0.93), or year (F_1,24_ = 0.9; *p* = 0.35) on tadpole acclimation (β) to NaCl (for details on β (Beta coefficient) calculations and figures, see Supplementary Materials Figures a2, a3, a4, a5, a6, a7, a8 and a9). There was also no significant interaction between Cohort*NaCl-acclimation (F_1,24_ = 1.2; p = 0.93), Year* NaCl-acclimation (F = 0.09; *p* = 0.76), or Year*NaCl-acclimation*Cohort (F_1,24_ = 1.1; *p* = 0.31). In contrast, we did find a significant interaction between Cohort*year (F_1,24_ = 4.8; *p* = 0.038). To better understand this interaction, we conducted Bonferroni-corrected pairwise comparisons. In 2018, we found a significant effect of cohort (F_1,24_ = 4.4; *p* < 0.001) on the ability for tadpoles from earlier vs. later-breeding cohorts to acclimate to higher concentrations of NaCl. Tadpoles from earlier-breeding cohorts reared in either 0.5 g L^− 1^ or 1 g L^− 1^ NaCl had lower β -values compared to tadpoles from later breeding cohorts (Fig. 7). This indicates that tadpoles from earlier breeding cohorts that were reared in 0.5 g L^− 1^ or 1 g L^− 1^ became more tolerant to higher concentrations of NaCl (negative β) whereas tadpoles from later-breeding cohorts became less tolerant to higher concentrations of NaCl (positive β).

## Discussion

Anthropogenic activities have serious consequences for species’ survival, particularly those species directly affected by both climate change and environmental contaminants. Here, we evaluated how breeding phenology (earlier versus later-breeding cohorts) and a common environmental pollutant interact to affect a widespread spring-breeding amphibian. At a single study region over 2 years, several populations of *R. sylvatica* began breeding activities early in response to spring-like temperature and precipitation cues; other populations did not breed until later, after the year’s final severe winter weather events were completed. In both years, when reared in the highest NaCl acclimation treatment, the earlier-breeding cohort produced smaller offspring than the later-breeding cohort. Interestingly, the effect of breeding phenology on tadpole mass seemed to be exacerbated as NaCl levels increased in the rearing environment. Although we do not have data for the earlier cohort of 2018, comparisons between the earlier and late cohorts of 2019 show no difference in their mass. However, when reared at an intermediate concentration of NaCl (0.5 g L^− 1^), the earlier cohort of 2019 produced significantly smaller individuals than that of the later-breeding cohort of the same year. Finally, when reared in the highest NaCl acclimation treatment (1 g L^− 1^ NaCl), the offspring of the later-breeding cohorts of 2018 and 2019 weighed ~ 37% and ~ 40% more on average, respectively, than offspring of the earlier-breeding cohorts within their respective years. Consistent with other pollutant studies, this suggests that the interactive effect of phenology and NaCl on mass is likely dose dependent [11].

We additionally found that wood frog tadpoles from the later-breeding cohort of 2018 were less tolerant of NaCl than tadpoles from the earlier-breeding cohort of the same year. While direct exposure to cold temperatures has been documented to lower immune responses [16, 54], reduce larval growth and development [63], and interact synergistically with other stressors [57], it is important to note that in this study, tadpoles were not directly exposed to the cold temperatures of the parental environment. Thus, the reduced baseline tolerance to NaCl of the late breeding cohort may reflect cross-generational consequences of parental exposure to suboptimal temperatures [51]. For example, Piiroinen et al. [51], found that exposure of Colorado potato beetles (*Leptinotarsa decemlineata*) to cold stress resulted in deleterious cross-generational effects, where offspring of cold-stressed parents were less tolerant of pyrethroid insecticides than those whose parents were reared at more optimal temperatures. Under climate change, we expect wildlife to contend with increasingly variable temperatures that can influence breeding phenology as well as facing challenges associated with pollutant exposure [45]. As such, our findings underscore the importance of evaluating the consequences of phenological shifts in concert with other stressors, such as NaCl exposure.

To better cope with contaminants in their environment, some individuals can increase their future tolerance to pollutants following an acclimation period to sublethal concentrations of the pollutant. Consistent with past work [67], we demonstrate that some populations of wood frogs are also capable of inducing increased tolerance to NaCl following exposure to NaCl early in life as embryos and hatchlings. Though previous research demonstrates that wood frogs can induce increased tolerance to pesticides [34], to our knowledge, this is the first evidence of NaCl-acclimation in this species. Whether this phenomenon is broadly generalizable across toxicants is still unknown. Though there is some evidence that wood frog populations that can induce tolerance to one pollutant are capable of inducing cross-tolerance to pollutants that share similar modes of action [33]. Interestingly, the ability for wood frogs to acclimate to NaCl differed between earlier versus later-breeding cohorts in 2018. Specifically, the later-breeding cohort of 2018 whose parents experienced a freezing event prior to breeding gave rise to offspring that were less capable of acclimating to NaCl exposure compared to the earlier-breeding cohort of the same year whose parents did not experience such events. We found that this pattern held regardless of the sublethal NaCl concentration at which tadpoles were exposed to prior to lethal exposure (0.5 or 1 g L^− 1^ NaCl). While the mechanisms underlying the reduced ability of the later-breeding cohort to acclimate to NaCl exposure were beyond the scope of this study, several studies suggest that amphibian acclimation to saline conditions is related to shifts in stress hormones, such as corticosterone (CORT) [59]. Extreme weather events, such as freezing temperatures, can upregulate CORT production in wildlife [10, 66], which can then be passed on to offspring [28]. While the expression of stress hormones contributes to addressing short term challenges, chronically elevated CORT is often associated with negative fitness effects [55]. Thus, future work might evaluate whether the reduced ability of the later-breeding cohort of 2018 to acclimate to NaCl exposure could be due to cross-generational fitness costs of parental expression of stress hormones. Collectively, while there is strong evidence demonstrating that amphibians can acclimate to diverse pollutants, this study suggests that considering parental environmental conditions may be critical to understanding the relative importance of acclimation as a mechanism for population persistence in the face of global change.

Interestingly, we found that the influence of breeding phenology (earlier vs. late) on baseline tolerance and acclimation differed between years. Temperature averages and variability throughout the two winters of our study can help to explain the differences in NaCl baseline tolerance and acclimation in 2018 and 2019. The later-breeding *R. sylvatica* cohort in 2018 experienced a more severe winter weather event than the later-breeding cohort in 2019 (5 versus 2 days of freezing temperatures in 2018 and 2019, respectively). The later-breeding cohort in 2018 therefore likely experienced greater physiological stress from the freeze than the later-breeding cohort in 2019; there are costly physiological responses to low temperatures [19, 63] and maternal stress is often transferred to developing eggs [18, 62] in a variety of herpetofauna. The higher level of stress experienced by the later-breeding cohort in 2018 may be one potential cause of reduced offspring baseline tolerance to NaCl compared to that in 2019. The difference in offspring NaCl tolerance (following NaCl acclimation) from earlier and later-breeding cohorts likewise reflects the pattern shown in the baseline tolerance: the offspring of later breeders were more susceptible than those of earlier breeders in 2018, whereas the 2019 earlier- and later-breeders’ offspring did not differ in susceptibility. Our findings suggest that small shifts in temperature in the parental environment may impact the viability of offspring in salinized systems. As shifting temperature regimes are likely to continue under global climate change and subsequently increase NaCl pollution across some regions of the globe [5, 41], our findings suggest a need for cross-generational studies to identify whether the patterns between earlier and late cohort survival following NaCl exposure seen here were indeed a product of freezing temperatures in the parental environment.

In this system, breeding timing (earlier vs. late) does not appear to be a locally adapted trait. Instead, it is well established that breeding behavior in wood frogs is induced by environmental cues [7]. More specifically, in the system we tested, in some breeding seasons (i.e., years with high variability in environmental cues), wood frog breeding times across populations are variable often splitting into earlier vs. later breeding populations [32, 34]. In contrast, in other years, these same wood frog populations may all breed on the same day [35]. The differential breeding times amongst and within wood frog populations across years used in our study are in line with those of other species, where differential breeding phenology has been posited to be due to differences in microclimate and/or weather [40, 44]. Importantly, as seen in this study, even within a population, there is evidence of annual variation in breeding time. Indeed, for the three populations (MIN, SKN, and MAL; Fig. 1), wood frogs differed in whether they laid earlier or later between 2018 and 2019. Because of this switch in relative breeding phenology between years, we were able to compare NaCl tolerance in early vs. later-breeding cohorts within a particular population. Consistent with the overall conclusions across populations, we found that tadpoles of earlier-breeders were more tolerant of NaCl than tadpoles of late-breeders even while holding population constant (SI- Figure A10). Collectively, this suggests that the early-late phenotypic differences in NaCl tolerance seen between the 2 years of study are unlikely to be completely due to underlying population-specific traits.

Lastly, we found that the conductivity of natal pond sites utilized by the wood frog populations used in our experiments differed. For some populations of amphibians, natal pond conductivity is associated with tolerance to NaCl exposure [8, 9, 30]. However, our correlation analyses revealed that natal pond conductivity was not a causal factor for the differential tolerance to NaCl seen in our study populations. Further, as eggs were removed from natal ponds ≤48 h following oviposition and were then placed immediately into filtered well water, the direct effects of natal pond environment on wood frog embryos were likely limited. Given the lack of correlation between pond conductivity and NaCl tolerance, along with the limited exposure of embryos to the natal environment, this altogether suggests the main contributor to the responses seen in our study were due to differences in breeding phenology and/or temperature in the parental environment.

### Future considerations

Our experimental design was highly conservative. By removing eggs from vernal ponds following breeding and rearing them in a temperature-controlled common garden environment, developing embryos from the early-breeding cohorts were protected from extreme low temperatures during the freeze. Thus, although offspring from the early-breeding cohort may have enhanced tolerance to NaCl compared to the later-breeding cohort, we did not account for the fact that these embryos likely would have experienced reduced growth and increased mortality in freezing ponds. In addition, the average temperature in the winter of 2019 was 1.03 °C warmer than that of 2018, and 2019 populations experienced an earlier phenological shift (i.e., both earlier and late breeders began breeding ~ 1 week earlier in 2019 than their counterparts in 2018). Warmer winter weather and earlier breeding (as documented at our sites in 2019) results in colder developmental temperatures for *R. sylvatica* larvae and corresponding reduced rates of development [7]. Overall, had offspring been reared in a field setting, it is likely that a) earlier cohorts in 2018 and 2019 would have experienced greater freeze-related mortality, and b) all larvae in 2019 would have experienced slowed development and exposure to stressful (cold) conditions. The consequences of these freezing conditions are evaluated in a companion paper [63]. Taken together, our data suggests that freezes following false springs (exacerbated by warmer average winter temperatures), particularly when combined with chemical stressors, are likely to be a major concern for spring breeding amphibian populations. Despite some debate on climate change’s direct role in amphibian extinctions [17], amphibians are increasingly identified as being highly vulnerable to climate change [23]. We suggest that the interactive effect of climate-induced phenological shifts and environmental contaminants is an underexplored and direct threat to amphibian populations.

## Supplementary Information

**Additional file 1: Supplementary Methods and Results.** Creation of salt solutions. Collection, analysis and results of temperature data. **Table 1.** Coordinates for wood frog populations. **Table 2.** Sample size of wood frog tadpoles that were weighed within each respective breeding cohort (early or late), year (2018 or 2019) and NaCl acclimation treatment (0, 0.5 or 1 g L^-1^ NaCl) combination. **Figure A1.** Natal pond conductivity for early vs. late breeding cohorts in 2018 and 2019. Different letters represent significance at *p* <0.05. **Figure A2.** Survival curves of tadpoles from seven populations of wood frogs in the early breeding cohort in 2018. The blue line represents tadpole tolerance to 8 g L^-1^ when not placed in an acclimation treatment. The red line represents tadpole tolerance to 8 g L^-1^ when reared in 0.5 g L^-1^ NaCl from the egg to tadpole stage. **Figure A3.** Survival curves of tadpoles from seven populations of wood frogs in the late breeding cohort in 2018. The blue line represents tadpole tolerance to 8 g L^-1^ when not placed in an acclimation treatment. The red line represents tadpole tolerance to 8 g L^-1^ when reared in 0.5 g L^-1^ NaCl from the egg to tadpole stage. **Figure A4.** Survival curves of tadpoles from seven populations of wood frogs in the early breeding cohort in 2018. The blue line represents tadpole tolerance to 8 g L^-1^ when not placed in an acclimation treatment. The red line represents tadpole tolerance to 8 g L^-1^ when reared in 1 g L^-1^ NaCl from the egg to tadpole stage. **Figure A5.** Survival curves of tadpoles from seven populations of wood frogs in the late breeding cohort in 2018. The blue line represents tadpole tolerance to 8 g L^-1^ when not placed in an acclimation treatment. The red line represents tadpole tolerance to 8 g L^-1^ when reared in 1 g L^-1^ NaCl from the egg to tadpole stage. **Figure A6.** Survival curves of tadpoles from five populations of wood frogs in the early breeding cohort in 2019. The blue line represents tadpole tolerance to 8 g L^-1^ when not placed in an acclimation treatment. The red line represents tadpole tolerance to 8 g L^-1^ when reared in 0.5 g L^-1^ NaCl from the egg to tadpole stage. **Figure A7.** Survival curves of tadpoles from five populations of wood frogs in the late breeding cohort in 2019. The blue line represents tadpole tolerance to 8 g L^-1^ when not placed in an acclimation treatment. The red line represents tadpole tolerance to 8 g L^-1^ when reared in 0.5 g L^-1^ NaCl from the egg to tadpole stage. **Figure A8.** Survival curves of tadpoles from five populations of wood frogs in the early breeding cohort in 2019. The blue line represents tadpole tolerance to 8 g L^-1^ when not placed in an acclimation treatment. The red line represents tadpole tolerance to 8 g L^-1^ when reared in 1 g L^-1^ NaCl from the egg to tadpole stage. **Figure A9.** Survival curves of tadpoles from five populations of wood frogs in the late breeding cohort in 2019. The blue line represents tadpole tolerance to 8 g L^-1^ when not placed in an acclimation treatment. The red line represents tadpole tolerance to 8 g L^-1^ when reared in 1 g L^-1^ NaCl from the egg to tadpole stage. **Figure A10.** Survival curves of tadpoles from three populations of wood frogs. The blue line represents the tolerance of tadpoles in the early-breeding cohort to NaCl. The red line represents the tolerance of tadpoles in the late-breeding cohort to NaCl.

## Data Availability

Data associated with this manuscript can be found at Binghamton University’s Open Repository (https://orb.binghamton.edu/bio_students/4/).
