# Supplementary material for: Amphibian breeding phenology influences offspring size and response to a common wetland contaminant
Source: Front Zool. 2021 Jun 25;18:31. doi: 10.1186/s12983-021-00413-0 (PMC8228996; doi:10.1186/s12983-021-00413-0)
Supplement: Supplementary file 1 — Additional file 1: Supplementary Methods and Results. Creation of salt solutions. Collection, analysis and results of temperature data. Table 1. Coordinates for wood frog populations. Table 2. Sample size of wood frog tadpoles that were weighed within each respective breeding cohort (early or late), year (2018 or 2019) and NaCl acclimation treatment (0, 0.5 or 1 g L-1 NaCl) combination. Figure A1. Natal pond conductivity for early vs. late breeding cohorts in 2018 and 2019. Different letters represent significance at p <0.05. Figure A2. Survival curves of tadpoles from seven populations of wood frogs in the early breeding cohort in 2018. The blue line represents tadpole tolerance to 8 g L-1 when not placed in an acclimation treatment. The red line represents tadpole tolerance to 8 g L-1 when reared in 0.5 g L-1 NaCl from the egg to tadpole stage. Figure A3. Survival curves of tadpoles from seven populations of wood frogs in the late breeding cohort in 2018. The blue line represents tadpole tolerance to 8 g L-1 when not placed in an acclimation treatment. The red line represents tadpole tolerance to 8 g L-1 when reared in 0.5 g L-1 NaCl from the egg to tadpole stage. Figure A4. Survival curves of tadpoles from seven populations of wood frogs in the early breeding cohort in 2018. The blue line represents tadpole tolerance to 8 g L-1 when not placed in an acclimation treatment. The red line represents tadpole tolerance to 8 g L-1 when reared in 1 g L-1 NaCl from the egg to tadpole stage. Figure A5. Survival curves of tadpoles from seven populations of wood frogs in the late breeding cohort in 2018. The blue line represents tadpole tolerance to 8 g L-1 when not placed in an acclimation treatment. The red line represents tadpole tolerance to 8 g L-1 when reared in 1 g L-1 NaCl from the egg to tadpole stage. Figure A6. Survival curves of tadpoles from five populations of wood frogs in the early breeding cohort in 2019. The blue line represents tadpole tolerance to 8 g L-1 [file 12983_2021_413_MOESM1_ESM.docx]

Supplementary Information for:

“Amphibian breeding phenology influences offspring size and response to a common wetland contaminant”

Authors: Nicholas Buss, Lindsey Swierk and Jessica Hua

Corresponding author: Nicholas Buss; nickrbuss@gmail.com

**Supplementary Methods and Results**

*Creation of salt solutions*

To create the salt solutions used in the experiment, we added laboratory grade NaCl (> 99 % NaCl, Baker Scientific) dissolved in aged well-water to produce concentrations of 0, 0.5, and 1 g L^-1^ and 8 g L^-1^ NaCl, respectively. We used a multi-parameter sonde (Xylem Analytics, Ohio, USA) to measure added NaCl into solution at the time of making the salt treatments. Background levels of salinity in our aged well-water controls were 0.034 g Cl^-^ L^-1^.

*Collection, analysis and results of temperature data*

We downloaded average temperatures in 2018 and 2019 from December 1 through April 30th from Wunderground.com. To compare the average temperatures in 2018 vs. 2019 during this time period we conducted Analysis of variance. We found a significant difference in average temperature between 2018 and 2019 (F = 4.8, p = 0.03; Figure 5). During this time period, the average temperature in 2018 was 83.5% lower than in 2019 (average difference of 1.8°C).

**Supplementary tables and figures**

Table 1. Coordinates for wood frog populations

| **Population** | **Coordinates** |
| --- | --- |
| SQR | 41°50.5′ N, 80°14.4’ W |
| BJ | 41°39.9′ N, 80°30.7’ W |
| LOG | 41°58.1′ N, 79°36.1' W |
| SKN | 41°59.929' N, 79° 46.501' W |
| MIN | 41°41.2’ N, 80°25.6’ W |
| MAL | 41° 41.507' N, 80° 30.085' W |
| TRL | 41°34.1′ N, 80°27.1' W |
| BOW | 41°55.6′ N, 79°48.2' W |
| HOP | 41°52.1′ N, 80°28.0′ W |
| SEW | 41° 39.041' N, 80° 25.756' W |
| RR | 41°36.4′ N, 80°22.9' W |
| BOR | 41°55.2′ N, 80°1.9' W |
| RMD | 41°43.9' N, 79°58.5' W |
| ROA | 41°53.1′ N, 79°36.3' W |

Table 2. Sample size of wood frog tadpoles that were weighed within each respective breeding cohort (early or late), year (2018 or 2019) and NaCl acclimation treatment (0, 0.5 or 1 g L^-1^ NaCl) combination.

| **Cohort, year, and NaCl acclimation treatment** | **Sample size** |
| --- | --- |
| Early 2018  (0 g L^-1^) | N/A |
| Late 2018  (0 g L^-1^) | 60 |
| Early 2019  (0 g L^-1^) | 50 |
| Late 2019  (0 g L^-1^) | 50 |
| Early 2018  (0.5 g L^-1^) | 43 |
| Late 2018  (0.5 g L^-1^) | 58 |
| Early 2019  (0.5 g L^-1^) | 61 |
| Late 2019  (0.5 g L^-1^) | 39 |
| Early 2018  (1 g L^-1^) | 70 |
| Late 2018  (1 g L^-1^) | 70 |
| Early 2019  (1 g L^-1^) | 50 |
| Late 2019  (1 g L^-1^) | 42 |

Figure A1. Natal pond conductivity for early vs. late breeding cohorts in 2018 and 2019. Different letters represent significance at p <0.05.

B = -0.37

P = 0.31

B = -0.003

P = 0.993

B = -1.3

P = 0.007

B = 0.38

P = 0.31

B = -0.8

P = 0.047

B = -0. 52

P = 0.18

B = -0.25

P = 0.5

Figure A2: Survival curves of tadpoles from seven populations of wood frogs in the early breeding cohort in 2018. The blue line represents tadpole tolerance to 8 g L^-1^ when not placed in an acclimation treatment. The red line represents tadpole tolerance to 8 g L^-1^ when reared in 0.5 g L^-1^ NaCl from the egg to tadpole stage.

B = 0.35

P = 0.35

B = -0.09

P = 0.82

B = 0.1

P = 0.79

B = 0.54

P = 0.16

B = 0.28

P = 0.47

B = -0.13

P = 0.74

B = -0.12

P = 0.74

Figure A3: Survival curves of tadpoles from seven populations of wood frogs in the late breeding cohort in 2018. The blue line represents tadpole tolerance to 8 g L^-1^ when not placed in an acclimation treatment. The red line represents tadpole tolerance to 8 g L^-1^ when reared in 0.5 g L^-1^ NaCl from the egg to tadpole stage.

B = -1.7

P = 0.66

B = -0.42

P = 0.46

B = -0.75

P = 0.08

B = -1.4

P = 0.003

B = -0.33

P = 0.38

B = -0.35

P = 0.35

Figure A4: Survival curves of tadpoles from seven populations of wood frogs in the early breeding cohort in 2018. The blue line represents tadpole tolerance to 8 g L^-1^ when not placed in an acclimation treatment. The red line represents tadpole tolerance to 8 g L^-1^ when reared in 1 g L^-1^ NaCl from the egg to tadpole stage.

B = 0.09

P = 0.81

B = -0.11

P = 0.79

B = 0.56

P = 0.16

B = 0.18

P = 0.65

B = 1.1

P = 0.012

B = -0.92

P = 0.04

B = 0.19

P = 0.61

Figure A5: Survival curves of tadpoles from seven populations of wood frogs in the late breeding cohort in 2018. The blue line represents tadpole tolerance to 8 g L^-1^ when not placed in an acclimation treatment. The red line represents tadpole tolerance to 8 g L^-1^ when reared in 1 g L^-1^ NaCl from the egg to tadpole stage.

Figure A6: Survival curves of tadpoles from five populations of wood frogs in the early breeding cohort in 2019. The blue line represents tadpole tolerance to 8 g L^-1^ when not placed in an acclimation treatment. The red line represents tadpole tolerance to 8 g L^-1^ when reared in 0.5 g L^-1^ NaCl from the egg to tadpole stage.

B = 0.54

P = 0.25

B = 0.46

P = 0.43

B = -0.13

P = 0.77

B = -0.38

P = 0.31

B = -0.14

P = 0.71

Figure A7: Survival curves of tadpoles from five populations of wood frogs in the late breeding cohort in 2019. The blue line represents tadpole tolerance to 8 g L^-1^ when not placed in an acclimation treatment. The red line represents tadpole tolerance to 8 g L^-1^ when reared in 0.5 g L^-1^ NaCl from the egg to tadpole stage.

B = 0.58

P = 0.12

B = 0.59

P = 0.12

B = -1.9

P = 0.62

B = 0.06

P = 0.87

B = 0.11

P = 0.77

Figure A8: Survival curves of tadpoles from five populations of wood frogs in the early breeding cohort in 2019. The blue line represents tadpole tolerance to 8 g L^-1^ when not placed in an acclimation treatment. The red line represents tadpole tolerance to 8 g L^-1^ when reared in 1 g L^-1^ NaCl from the egg to tadpole stage.

B = -0.23

P = 0.54

B = 0.46

P = 0.21

B = -0.50

P = 0.19

B = -0.19

P = 0.64

Figure A9: Survival curves of tadpoles from five populations of wood frogs in the late breeding cohort in 2019. The blue line represents tadpole tolerance to 8 g L^-1^ when not placed in an acclimation treatment. The red line represents tadpole tolerance to 8 g L^-1^ when reared in 1 g L^-1^ NaCl from the egg to tadpole stage.


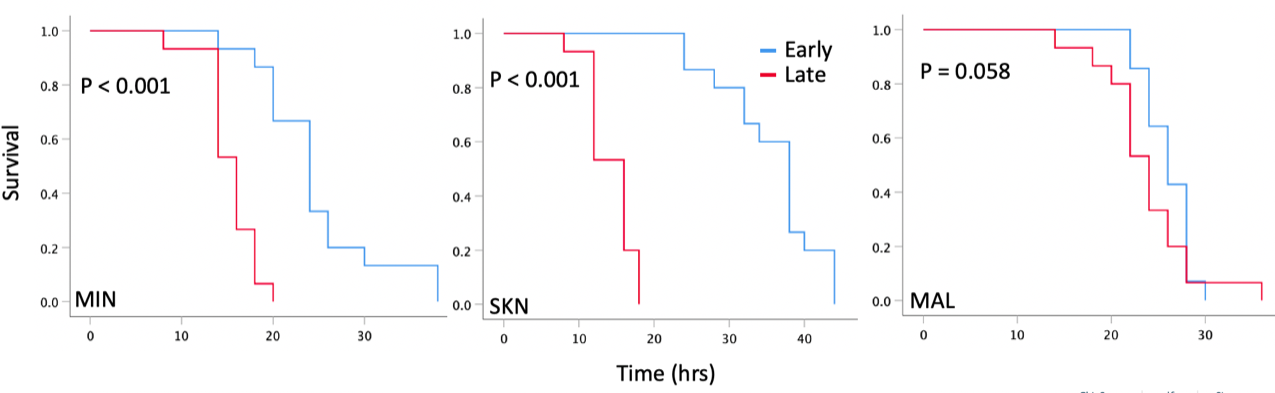


Figure A10: Survival curves of tadpoles from three populations of wood frogs. The blue line represents the tolerance of tadpoles in the early-breeding cohort to NaCl. The red line represents the tolerance of tadpoles in the late-breeding cohort to NaCl.
